# Supplementary material for: Epigenetic and physiological alterations in zebrafish subjected to hypergravity
Source: PLoS One. 2024 May 22;19(5):e0300310. doi: 10.1371/journal.pone.0300310 (PMC11111069; doi:10.1371/journal.pone.0300310)
Supplement: Attachment — Submitted filename: Response to reviewers_final_050224.docx [file pone.0300310.s001.docx]

**Comments to the Author**

1. Is the manuscript technically sound, and does the data support the conclusions?

The manuscript must describe a technically sound piece of scientific research with data that supports the conclusions. Experiments must have been conducted rigorously, with appropriate controls, replication, and sample sizes. The conclusions must be drawn appropriately based on the data presented.

Reviewer #1: Partly

Reviewer #2: Partly

2. Has the statistical analysis been performed appropriately and rigorously?

Reviewer #1: No

Reviewer #2: No

3. Have the authors made all data underlying the findings in their manuscript fully available?

The [PLOS Data policy](http://www.plosone.org/static/policies.action#sharing) requires authors to make all data underlying the findings described in their manuscript fully available without restriction, with rare exception (please refer to the Data Availability Statement in the manuscript PDF file). The data should be provided as part of the manuscript or its supporting information, or deposited to a public repository. For example, in addition to summary statistics, the data points behind means, medians and variance measures should be available. If there are restrictions on publicly sharing data—e.g. participant privacy or use of data from a third party—those must be specified.

Reviewer #1: Yes

Reviewer #2: No

4. Is the manuscript presented in an intelligible fashion and written in standard English?

PLOS ONE does not copyedit accepted manuscripts, so the language in submitted articles must be clear, correct, and unambiguous. Any typographical or grammatical errors should be corrected at revision, so please note any specific errors here.

Reviewer #1: Yes

Reviewer #2: No

5. Review Comments to the Author

Please use the space provided to explain your answers to the questions above. You may also include additional comments for the author, including concerns about dual publication, research ethics, or publication ethics. (Please upload your review as an attachment if it exceeds 20,000 characters)

**Reviewer #1**: This manuscript addresses the effects of hypergravity on developmental, behavioral, gene expression, and epigenetic changes in zebrafish. The authors showed significant hypermethylation of the genome and downregulation (not significant) of gene expression levels of three genes under hypergravity for 5 days. This study is important for understanding the effects of hypergravity in DNA methylation level, but needs to be modified before publication in Plos One.

1. Figure 4 and Table 1 show the identification of three locomotor aspects. It could be more easily understood if video data or other means were used to demonstrate these typical behaviors.

Thanks, for the comment. We have added new support information creating a repository of the videos of this manuscript available in the DigitalCSIC repository: <https://doi.org/10.20350/digitalCSIC/16106>.

which are the videos exhibiting every morphological characteristic observed in both the control and hypergravity groups of zebrafish larvae.

2. Figure 5 shows various phenotypes under hypergravity. Each is assumed to have different conditions, such as tissue inflammation and damage. Did the authors perform the histological analysis? And how did the authors evaluate gene expression and genomic methylation in zebrafish exhibiting these various phenotypes?

Thanks for pointing this out. Unfortunately, histological analysis was not performed because we used the whole larvae for the DNA and RNA extractions, and therefore no more tissues were available.

Gene expression and methylation analysis were randomly conducted within the groups; 15 samples were ethologically evaluated from each experiment, and 10 of these were randomly selected for the aforementioned studies. We decided not to select any specific phenotype as this could bias the result. However, after evaluating our raw data from the experiment, we have created the following table with the selected phenotypes of those larvae used for molecular analysis.

| **Teratologies** | **Control** | **Hypergravity** |
| --- | --- | --- |
| Normal body shape | 12 | 0 |
| Overall body deformation | 1 | 6 |
| Tail anomalies | 1 | 0 |
| Body curvature | 1 | 9 |

3. The authors attempt to show in only one figure (Figure 6) the over methylation of the zebrafish genome under 5 days of hypergravity. The results of the comprehensive analysis should be evaluated from various angles.

The reviewer is right with the suggestion. In the current form of the study, we aimed to explore, for the first time, the epigenetic changes that hypergravity could trigger in zebrafish larvae. As the findings are promising, future studies in our lab will be addressed towards a better comprehension of the epigenome modifications from various angles. We are currently conducting RNA-seq from a similar experiment hoping that they will untangle molecular events by gene expression modifications.

4. Figure 7 describes that there was a decrease in the expression of three genes, but it was not significant. Were significant decreases in gene expression also seen in other genes?

We did not evaluate other genes because we wanted to assess these important genes related to methylation. Based on our experience in the lab, we used them as epimarkers for gene expression studies related to methylation alterations (Ribas et al 2017 https://doi.org/10.1186/s13072-017-0168-7; Valdivieso et al 2023 DOI: 10.3390/ijms242116002). However, as previously mentioned, we are working in the lab on RNA-sequencing technologies and we hope we will be able to identify differential gene expression.

5. Are there any specific sites of DNA methylation under hypergravity conditions?

For the current study on Global DNA methylation (Zymo Research), the methodology utilized unfortunately lacks the capability to discern specific methylation sites. Despite this limitation, we are actively engaged in the experimentation and evaluation of an alternative technique for our upcoming study. In particular, we are exploring the application of the MinION, a cutting-edge technology that holds promise for providing insights into the methylation patterns. This innovative approach is expected to enhance our ability to pinpoint and analyze specific methylation sites with greater precision, thereby contributing to a more comprehensive understanding of the underlying molecular mechanisms.

6. Is "Hipergravity" misspelled in figure 6?

Thanks, the figure has been amended

Reviewer #2: In this study, Salazar et al. studied the effects of hypergravity on development, behavior, gene expression, and epigenetic alterations in zebrafish. The authors revealed that hypergravity decreased survival, but not hatching rate at 2 days post fertilization. Hypergravity induced physiological and morphological changes including posture, movement frequency, and swimming behavior. Moreover, hypergravity increased DNA methylation and tended to decrease epigenetic-related genes, such as dnmt1, dnmt3, and tet1, without statistical significance in the zebrafish larvae. However, there are several issues with the manuscript. The details are attached below.

Major points
1. Please provide a rationale for used 3 g hypergravity condition in the present study.

Thanks for your comments. We performed two pilot studies before selection 3g hypergravity as the experimental conditions for our experiments. The explanation concerning the selection of 3g have been incorporated into the manuscript.

2. The detailed methods for analysis of qPCR should be added in Methods section. Did the authors calculated data using ΔΔCt methods? Which endogenous control was used?

Yes, we used the ΔΔCt method with two endogenous genes, RPL13A (Ribosomal Protein L13A) and EFα (Elongation Factor α). We have already amended the text accordingly.

3. Although Table 2 showed primer sequence for ef1α and rpl13a, the authors did not show those quantitative data.

These genes were the endogenous controls that were used to normalize the target genes to evaluate the expression making a qualitative study. This method allows to study of gene expression based on qualitative data all the values normalized to their endogenous genes to later compare them with their corresponding controls. Thus, these analyses are not quantitative, in compared to using a specific probe for each gene by qPCR. We hope that adding information to the text as mentioned in the previous comment makes the analysis clearer.

4. Fig. 6: According to figure legend, data were expressed as boxplot with thick line indicating median. However, figure 6 were not expressed boxplot. Please correct figure 6 and its figure legend.

Thanks, the text in the legend has been amended accordingly.

5. Statistical analysis should be correct. Although the authors revealed that the Shapiro-Wilk test was used to check for normality in the Methods section, normality was evaluated with a Kolmogorov-Smirnov test in Fig. 3. The authors used a student t-test for assessment of differences in Fig. 6, but there is no description regarding t-test in the Methods section. It is not clear which statistics were used in Fig. 7.

Thanks, the text has been amended accordingly.

6. PLOS authors have the option to publish the peer-review history of their article ([what does this mean?](https://journals.plos.org/plosone/s/editorial-and-peer-review-process#loc-peer-review-history)). If published, this will include your full peer review and any attached files.


If you choose “no”, your identity will remain anonymous but your review may still be made public.


**Do you want your identity to be public for this peer review?** For information about this choice, including consent withdrawal, please see our [Privacy Policy](https://www.plos.org/privacy-policy).

Reviewer #1: **Yes:** Masahiro Chatani

Reviewer #2: No
